# Liver Transplant From a Deceased Donor With Cystinosis: A Case Report

**DOI:** 10.1002/jmd2.12467

**Published:** 2025-01-09

**Authors:** Raeda Taj, Kim Ng, Sanmati R. Cuddapah, Elizabeth B. Rand, Melissa Bleicher, Sandra Amaral, Maarouf A. Hoteit, Rajendar K. Reddy, Eyob Feyssa, Emma E. Furth, Kim M. Olthoff, Samir Abu‐Gazala, Matthew H. Levine, Frederick Vyas, Peter L. Abt

**Affiliations:** ^1^ Department of Surgery, Transplant Division Hospital of the University of Pennsylvania Philadelphia Pennsylvania USA; ^2^ Division of Human Genetics Children's Hospital of Philadelphia Philadelphia Pennsylvania USA; ^3^ Department of Pediatrics, Division of Gastroenterology, Hepatology and Nutrition Children's Hospital of Philadelphia Philadelphia Pennsylvania USA; ^4^ Division of Nephrology, Department of Medicine Hospital of the University of Pennsylvania Philadelphia Pennsylvania USA; ^5^ Division of Nephrology, The Children's Hospital of Philadelphia, Department of Pediatrics and Department of Biostatistics, Epidemiology and Informatics Perelman School of Medicine, University of Pennsylvania Philadelphia Pennsylvania USA; ^6^ Division of Gastroenterology and Hepatology Hospital of the University of Pennsylvania Philadelphia Pennsylvania USA; ^7^ Department of Pathology and Laboratory Medicine Hospital of the University of Pennsylvania Philadelphia Pennsylvania USA

**Keywords:** cystinosis, inherited metabolic disorders, liver transplant

## Abstract

Many inherited metabolic disorders (IMD) are associated with end‐organ damage necessitating organ transplantation. Although utilization of deceased donors with history of IMD warrants caution, there may be circumstances under which such donors could be considered as suitable organ donor candidates. We present the first known report of liver transplantation from a deceased donor with cystinosis. The donor was a 20‐year‐old male with infantile cystinosis who had previously undergone two deceased donor kidney transplants. Unfortunately, he incurred cranial trauma, and after careful consideration of the metabolic consequences, his liver was deemed suitable for transplantation. The liver was successfully transplanted into a 65‐year‐old female recipient with hepatitis C (HCV) cirrhosis. The recipient is currently 12 months post‐transplant and experiencing good graft function without evidence of cystine crystals on liver biopsy. This case highlights that liver transplantation from donors with rare IMD can result in favorable outcomes. However, it is crucial to approach the use of such livers with caution. These transplants should be considered after a thorough assessment, ensuring that a comprehensive decision‐making process is in place to mitigate potential risks.

**Summary**

- This case report highlights the potential for successful liver transplantation from donors with cystinosis, offering valuable insights and considerations for expanding the donor pool with rare inherited metabolic disorders.

## 1 Introduction

**FIGURE 1:**
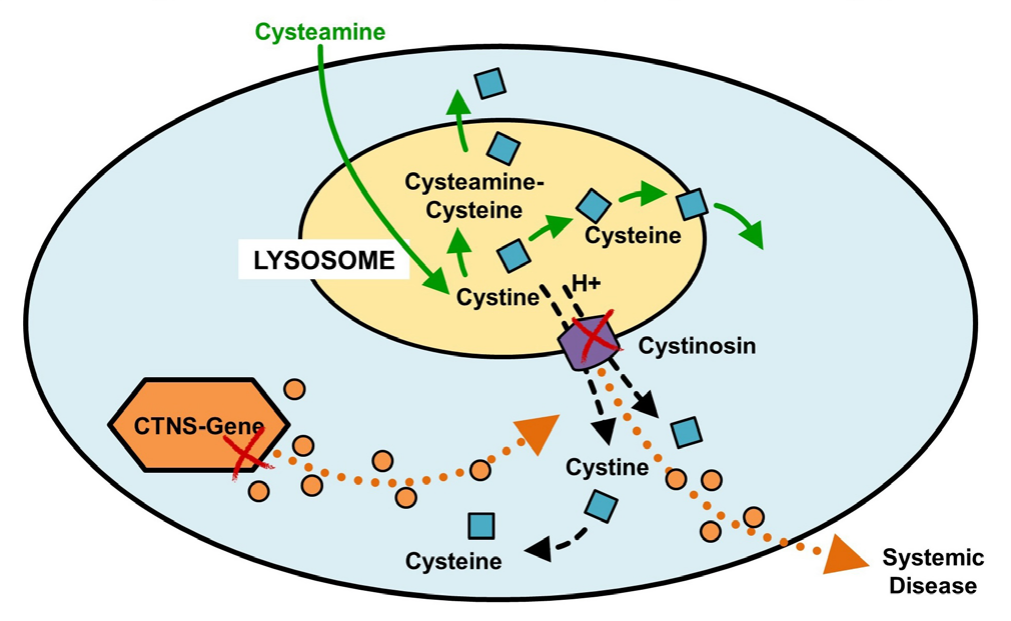
Cystinosis pathophysiology: Cystine exits the lysosome via cystinosin through cystine‐proton symport (black arrows). CTNS gene pathogenic variants impair cystinosin function leading to cystine crystal formation and accumulation within the lysosome (orange arrows). Cysteamine treatment induces a chemical reaction that converts cystine to cysteamine–cysteine and cysteine which exit the lysosome by bypassing the cystinosin transporter protein (green arrows).

**FIGURE 2:**
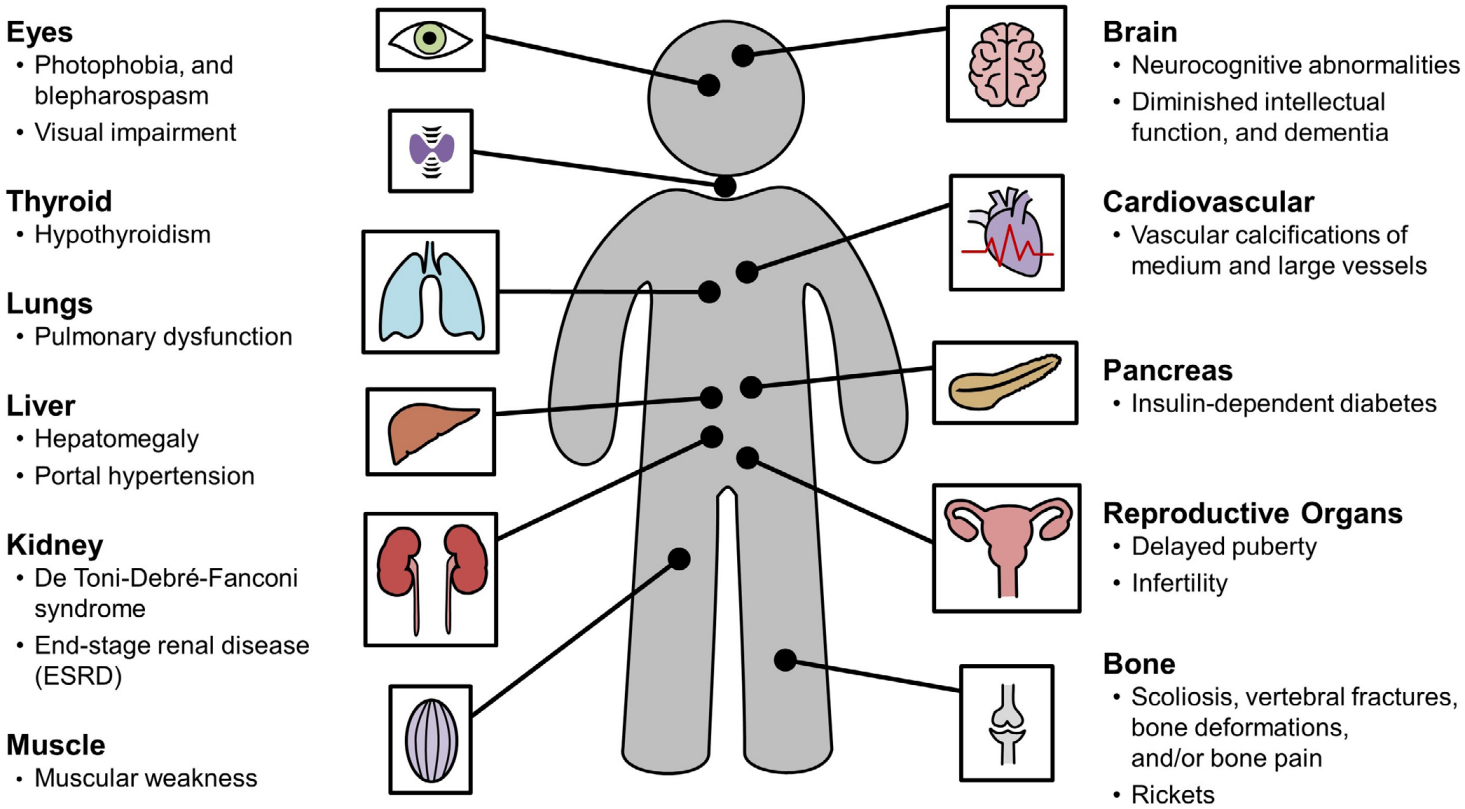
Clinical manifestations of Cystinosis.

Cystinosis is a rare metabolic autosomal recessive lysosomal storage disorder with an estimated incidence of 1 per 100 000 to 200 000 live births [1]. It is attributed to bi‐allelic pathogenic variants in the CTNS gene located on the short arm of chromosome 17 (p13), which encodes the lysosomal cystine transporter protein cystinosin. Defective cystinosin leads to impaired cystine transport from lysosomes to cystosol, resulting in cystine accumulation in lysosomes and crystal formation, and subsequent cellular dysfunction (Figure 1) [2]. Infantile nephropathic cystinosis is the most prevalent form of the disease, which affects multiple organs, with the kidneys and eyes being the most commonly involved (Figure 2) [3].

Renal involvement leads to renal Fanconi syndrome, driven by several pathogenic mechanisms, including proximal tubular cells (PTCs) dedifferentiation, impaired vesicular trafficking, defective mTOR signaling and autophagy [4]. It manifests as polyuria, glycosuria, phosphaturia, aminoaciduria, tubular proteinuria, growth retardation, and rickets. Eventually, leading to glomerular involvement and focal segmental glomerulosclerosis (FSGS), progressing to kidney failure by the age of 10 if untreated [1, 4]. Ocular involvement results from corneal crystal deposition causing photophobia, corneal erosions and loss of visual acuity [5].

Liver disease in individuals with cystinosis is a rare occurrence and is primarily attributed to the accumulation of cystine in Kupffer cells [6]. In cases of long‐term infantile nephropathic cystinosis, a specific form of sclerosing cholangitis may develop, which has demonstrated a favorable response to treatment with ursodeoxycholic acid [7]. It is not uncommon to observe hepatomegaly without liver dysfunction, which occurs in 40% of patients. The accumulation of cystine crystals activates Kupffer cells, prompting the release of vasoactive substances that induce vasoconstriction in the microcirculation, ultimately leading to portal hypertension [8].

There are three main diagnostic modalities for cystinosis. The gold standard is the detection of elevated intracellular cystine, specifically in granulocytes; additional diagnostic methods include the detection of corneal cystine crystals by slit lamp examination or molecular testing of *CTNS* showing bi‐allelic pathogenic variants [9, 10]. Patients typically undergo regular monitoring of cystine levels in granulocytes, which is aimed at assessing compliance with cysteamine treatment, and repeated eye examinations to monitor corneal disease.

The treatment of cystinosis consists of supportive treatment for Fanconi syndrome, and specific lifelong cystine‐depleting therapy using oral cysteamine [11]. There is strong evidence that early initiation and ongoing therapy with cysteamine are essential for delaying the progression to kidney failure, end‐organ damage, and extrarenal involvement [12, 13]. Given that the cornea is avascular, topical cysteamine eye drops are required to prevent corneal deposition [11]. We present the first known report of a deceased donor liver transplant from a prior kidney recipient with cystinosis.

## 2 Case Presentation

The donor was a 20‐year‐old male diagnosed with infantile cystinosis at age of two. He underwent *two deceased donor kidney transplants, the first at the age of five, which was complicated by* antibody mediated rejection *and graft failure, followed by a second transplant at the age of 20. Throughout his follow‐up years, he was maintained* on systemic cysteamine and cysteamine eyedrops, and his liver function tests (LFTs) were consistently normal. *Unfortunately, the donor succumbed to trauma resulting in* a 3.4 cm laceration to segment IVb of the liver with mild elevation of alanine transaminase (ALT: 131 U/L) and aspartate transaminase (AST: 191 U/L). After extensive discussion with metabolism and nephrology specialists, the liver was deemed biochemically suitable for transplantation. At the time of procurement, the liver was inspected and found to be of normal quality, albeit with the anticipated laceration. A procurement biopsy demonstrated 15% microvesicular steatosis, and no macrovesicular steatosis or fibrosis.

The recipient was a 65‐year‐old female with history of HCV‐related cirrhosis, Model of end stage liver disease (MELD) score of 17, complicated by ascites requiring frequent large volume paracentesis and muscle wasting. Additionally, the patient had declining kidney function with an *estimated glomerular filtration rate* (eGFR) of 38 mL/min, which did not qualify her for a simultaneous liver kidney (*SLK*) *transplant*. The patient and her family were informed about the donor's rare metabolic disorder, and the absence of long‐term outcome data. After a thorough discussion, they expressed their willingness to proceed. The patient underwent an uncomplicated orthotopic liver transplantation (OLT). The total cold ischemic time (CIT) was 209 min. A post perfusion liver biopsy revealed marked hemosiderin deposition in Kupffer cells without notable inflammation, steatosis, or fibrosis. The patient was initiated on intraoperative continuous renal replacement therapy (CRRT) and was eventually transitioned to *hemodialysis* (HD) due to the absence of renal recovery. The patient was discharged on post‐operative day 18.

**FIGURE 3:**
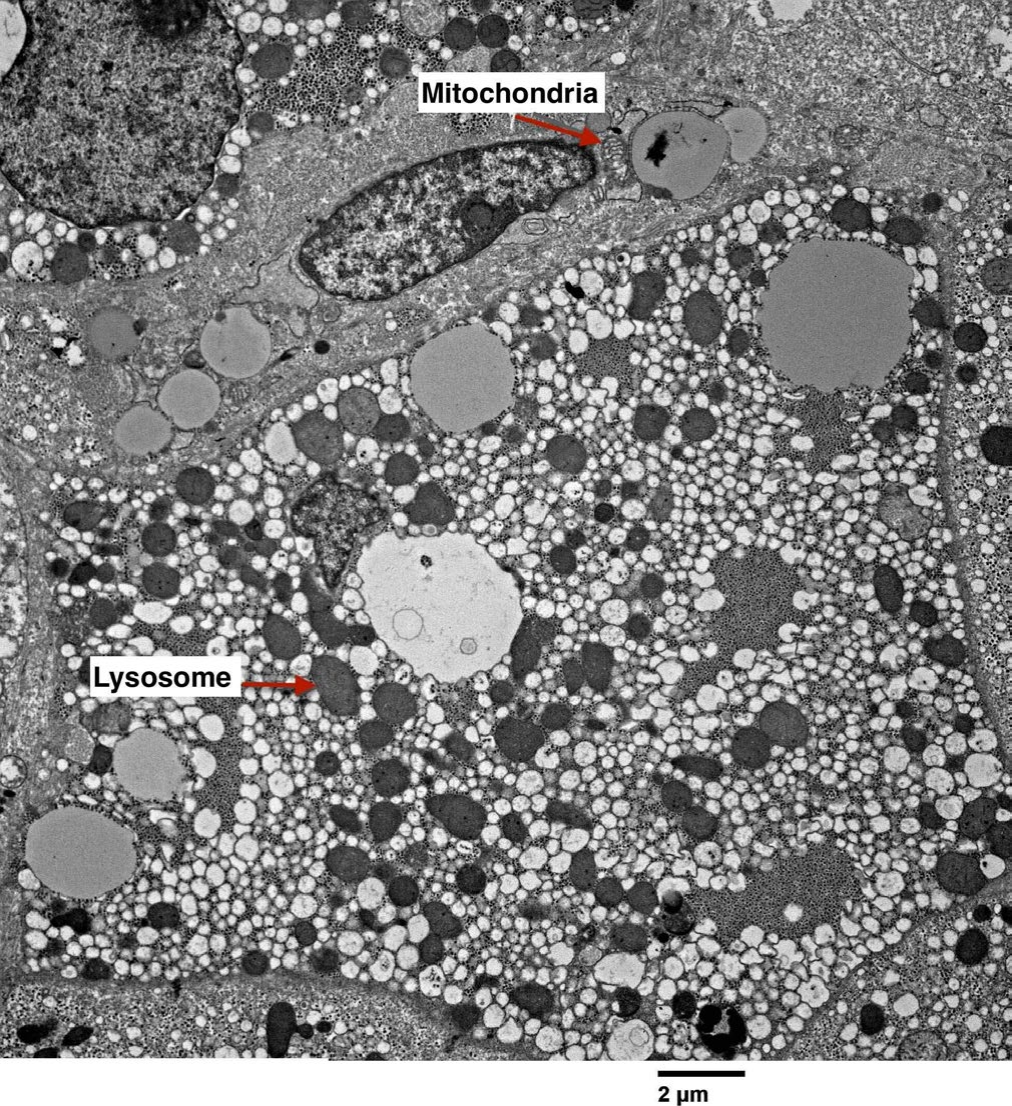
Liver biopsy electron microscopy shows normal morphology of hepatocytes and Kupffer cells in the sinusoidal space, with no crystal deposition detected.

A surveillance liver biopsy to detect cystine crystals was performed 6 months post‐transplant. While no chemical measurements of cystine content were conducted, the biopsy was prepared using electron microscopy (EM) with a standard glutaraldehyde technique on fresh liver tissue, and frozen section analysis was performed in two ways: (1) an unfixed section was directly cover‐slipped with phosphate‐buffered saline (PBS) and examined under polarized light microscopy, and (2) a briefly methanol‐fixed frozen section was examined similarly for polarizable crystalline structures. No cystine crystals were observed in the liver tissue (Figure 3). The biopsy showed minimal centrivenular fibrosis and hemosiderin deposition in Kupffer cells, suggesting that the liver is unlikely affected by cystinosis.

After extensive discussion with metabolism specialists, it was determined that cysteamine therapy would not be initiated and to repeat the liver biopsy 18 months post‐transplant. Patient remains well with normal liver function 12 months post‐transplant and is currently listed for a kidney transplant.

## 3 Discussion

The mismatch between the demand for organ transplantation and supply of organs necessitates consideration of all organ donation opportunities. Certain IMDs that are often indications for transplantation may offer opportunities for donation. For example, familial amyloid polyneuropathy (FAP) is an indication for both liver and heart transplantation. Individuals with FAP can be liver donors, recognizing the potential for long‐term neurologic complications in the recipient. Other rare metabolic disorders where the liver has been used for transplantation include hereditary hemochromatosis, Gilbert syndrome, factor VII deficiency, pseudoxanthoma elasticum, and maple syrup urine disease [14].

Thorough multidisciplinary evaluation is crucial to determine the suitability of transplanting a specific organ from a donor with an IMD. Factors to consider include the impact of the genetic changes on the specific organ intended for transplant, potential systemic adverse effects on the recipient from the transplanted organ and their timeline, available therapeutic options to mitigate the impact on the recipient, evaluation of the recipient's suitability for the organ, weighing alternative transplant options, assessing waitlist mortality risk and obtaining patient's informed consent.

In this liver transplant recipient, cystine accumulation is isolated to the liver, so other organs will not be affected since only the liver is transplanted. The progression of this patient's chronic kidney disease to end stage renal disease (ESRD) following OLT was likely a result of acute tubular necrosis and was not related to the donor's cystinosis. Traditional means of following a patient with cystinosis, such as monitoring granulocyte cystine levels are not diagnostically informative. LFTs could demonstrate elevation if crystals develop in the liver.

While no cystine crystals were observed on the liver biopsy, there remains a possibility that the liver could be affected over time. After extensive discussion with metabolism specialists, the decision was made not to initiate cysteamine therapy for the time being. Plan to continue regular liver surveillance, including periodic LFTs and repeat liver biopsy 18 months post‐transplant. We acknowledge that any potential liver involvement may take time to become evident in future biopsies.

There is an ongoing clinical trial investigating the possibility of a one‐time autologous transplantation of CTNS gene‐modified hematopoietic stem and progenitor cells (HSPCs) to provide a lifelong therapeutic solution in cystinosis that may eliminate the need for kidney transplantation and reduce long‐term complications [15]. As new innovative therapies for IMD emerge, there will be a shift in phenotypic characteristics of this rare donors pool resulting in unique considerations in managing the organ recipients.

## Author Contributions

R.T., K.N., S.R.C. and P.L.A. conceived and planned the study, E.B.R., M.B., S.A., M.A.H., R.K.R., E.F., and E.E.F. reviewed and reported the study. K.M.O., S.A.‐G., M.H.L., F.V. were involved in drafting and writing the manuscript. All authors contributed to reviewing the final manuscript.

## Consent

Informed consent was obtained from all patients for being included in the study.

## Conflicts of Interest

The authors declare no conflicts of interest.

## Data Availability

The data for this case report are based on clinial observations and are not publically available to ensure patient confidentiality. De‐identified data may be available upon request with appropriate approvals.
